# Clinical, humanistic, and economic burden of systemic lupus erythematosus in the Kingdom of Saudi Arabia

**DOI:** 10.1186/s12962-025-00678-w

**Published:** 2025-11-25

**Authors:** Ahmed Al-jedai, Hajer Al-Mudaiheem, Nayef Al Ghanim, Maysa Eshmawi, Ibrahim AlHomood, Pratik Dhopte, Rita Ojeil

**Affiliations:** 1 Colleges of Medicine and Pharmacy, Alfaisal University, Riyadh, Saudi Arabia; 2 Saudi Society of Clinical Pharmacy, Riyadh, Saudi Arabia; 3 King Saud Medical City, Riyadh, Saudi Arabia; 4 College of Medicine, Imam Mohammed Ibn Saud Islamic University, Riyadh, Saudi Arabia; 5 King Abdullah Medical College Complex, Jeddah, Saudi Arabia; 6 King Fahad Medical City, Riyadh, Saudi Arabia; 7 Carexso, Dubai, United Arab Emirates

**Keywords:** Systemic lupus erythematosus, Burden of disease, Autoimmunity, Kingdom of Saudi Arabia, Economic burden

## Abstract

**Introduction:**

Systemic Lupus Erythematosus (SLE) is an autoimmune disease characterized by abnormal immune system activity, primarily affecting the working-age population. The study aimed to evaluate SLE’s burden of disease from a societal perspective in the Kingdom of Saudi Arabia (KSA).

**Methods:**

The burden of disease model was developed using Microsoft Excel^®^ as the analytical tool for a one-year time frame. It was based on various inputs gathered through a literature review, input from key opinion leaders, and one-way sensitivity analysis, followed by local Delphi panel validation.

**Results:**

The overall economic burden of SLE proper, lupus nephritis, and lupus central nervous system (CNS) for one year amounted to SAR 46,187,056, SAR 78,776,030, and SAR 19,822,905, respectively. This condition resulted in 263.6 years lived with disability (YLD) and 251.1 years of life lost (YLL), resulting in a total of 514.7 disability-adjusted life years (DALYs), valued at SAR 58,768,258 for SLE proper. For lupus nephritis, the burden is 542.96 DALYs (232.12 YLD and 310.85 YLL), amounting to SAR 61,995,057 and 129.67 DALY (10.55 YLD and 119.12 YLL) valued at SAR 14,805,541 for lupus CNS. Event management cost was the primary contributor to the economic burden, followed by high drug acquisition cost, which also significantly adds to overall healthcare expenses. The introduction of biologics to the treatment of SLE is expected to curb the monetary losses due to the humanistic burden of the disease.

**Conclusion:**

To address the potential burden of SLE on the Saudi healthcare system, it is recommended to improve access to biologic therapies, enhance disease awareness, and implement targeted policy measures to support effective resource allocation.

**Supplementary information:**

The online version contains supplementary material available at 10.1186/s12962-025-00678-w.

## Key points

1. Systemic Lupus Erythematosus (SLE) is a chronic autoimmune disease with significant implications for the working-age population, yet data on its full burden in the Kingdom of Saudi Arabia (KSA) have been limited.

2. This study fills a critical evidence gap by quantifying the clinical, humanistic, and economic burden of SLE in KSA through a comprehensive societal perspective, integrating healthcare costs, productivity loss, and quality-of-life impacts.

3. Findings reveal that SLE imposes a substantial burden on the Saudi healthcare system and society, underscoring the need for improved access to biologics, enhanced disease awareness, and targeted policy interventions to inform HEOR and optimize resource allocation.

**Supplementary information:**

The online version contains supplementary material available at 10.1186/s12962-025-00678-w.

## Background

Systemic lupus erythematosus (SLE) is a chronic autoimmune disease that affects multiple organs, causing diverse symptoms and inflammation, with periods of relapses [1–4]. SLE is characterized by autoantibodies targeting nuclear and cytoplasmic genetic material. The exact cause is unknown, but genetics, the immune system, hormones, and the environment play crucial roles in disease activity [1]. The typical clinical manifestations of SLE include symptoms that can vary widely among individuals, ranging from mild to severe, and may include joint pain, skin rashes, pleuritis, pericarditis, renal or central nervous system (CNS) involvement, and hematologic abnormalities. Diagnosis is challenging due to the disease’s ability to mimic other conditions and requires both clinical and serologic criteria [5].

Globally, the incidence of SLE is 5.14 cases per 100,000 person-years, with approximately 0.40 million new cases diagnosed annually [2]. The worldwide prevalence of SLE is estimated to be 43.7 per 100,000 persons, affecting around 3.41 million people [2]. While lupus can affect people of all ages, it is more common in individuals aged 15 to 45 years [6]. The risk of premature mortality in SLE patients is twice that of healthy individuals [7]. Women are around ten times more likely to develop SLE than men, and the majority of cases occur during childbearing age [3]. Asians, Hispanics, and Blacks are at a higher risk of SLE compared to Caucasians [4].

Limited data exists on the prevalence of SLE in the Middle East, particularly in the Kingdom of Saudi Arabia (KSA) [5]. In Central KSA, the reported prevalence is around 19.28 per 100,000 people [8]. In Oman, the average occurrence of SLE is 38.8 per 100,000 individuals, with a range of ages 5 to 63 [9]. The crude incidence ratio of SLE in the United Arab Emirates (UAE) is 2.1 per 100,000 people [9].

Burden of disease (BOD) studies, also known as cost-of-illness (COI) analysis, track a disease’s clinical, economic, and humanistic impact on entire populations through illness and death rates (morbidity and mortality) [10]. These studies go beyond medical costs to capture the disease’s full impact, including patient well-being, healthcare needs, and economic burdens across various levels (national, regional, etc.). This knowledge is essential for prioritizing treatments and efficiently allocating resources within budget constraints [11, 12]. SLE places a significant strain on the healthcare system because of difficulties and delays in diagnosis, heterogeneity, and non-specificity of clinical presentation in the initial stage, higher disease activity, and SLE-related organ damage management [13]. People with SLE have a significantly worse chance of survival compared to the general population, with death rates 2–3 times higher [14]. Worldwide, infections and cardiovascular disease are the top causes of death for SLE patients [4]. A retrospective study in KSA (2001–2009) found an 8.2% mortality rate in 99 SLE patients. The leading causes of death were sepsis (62.5%), followed by ischemic heart disease and pulmonary embolism (both at 12.5%) [15].

In the KSA, several factors contribute to the disease burden and affect mortality and morbidity rates among SLE patients. These include a high prevalence of cardiovascular issues such as myocardial infarction and coronary artery disease [16], as well as neuropsychiatric symptoms like fear, altered sensations, cerebrovascular disease, and sleep disturbances [17]. Lupus flares and infections are the leading causes of hospitalization with intensive care unit (ICU) admissions in KSA, primarily due to acute respiratory distress syndrome and pulmonary hemorrhage [18].

In KSA, 68.4% of lupus flares, 20.4% of infections, and the presence of cutaneous manifestations and other end-organ impairments led to hospitalization [19]. These factors impose a high direct cost on SLE patients [19]. It is noteworthy that hospital admissions also raise the indirect costs of SLE [17]. In the UAE, indirect costs such as productivity loss due to SLE among adult-employed patients were estimated at AED 7 billion (USD 3.1 billion), and from a societal perspective, the cost per patient per year was AED 148,468 (USD 66,578) [20]. In the US, systemic lupus erythematosus (SLE) is a leading cause of hospital readmissions and imposes significant healthcare and prescription costs [21]. Patients with moderate or severe SLE incur higher direct costs when using biologics and glucocorticoids [22]. Additionally, SLE patients report three times greater physical function limitations compared to non-SLE individuals. The yearly direct total cost per SLE patient in the US ranges from USD 19,099 to 82,391 [23].

The humanistic burden [24] and intangible costs [25] added upon patients by a disease can be measured by health-related quality of life (HR-QOL). The QOL is consistently poorer in SLE patients than in healthy or chronic obstructive pulmonary disease or rheumatoid arthritis patients [26]. In a study conducted in the KSA, 18% of the studied SLE patients were found to have anxiety, and 20% had an absence of interest, indicating depression in SLE patients [27]. Anxiety and depression were significantly linked with a lengthier disease duration, joblessness, smoking, and the incidence of comorbidities [28]. In another study, a total of 62.1% were reported to have had trouble adhering to SLE medication. The severity of depressed mood was significantly correlated with disease activity (*p* = 0.003) [29]. Moreover, in their study, AlHomood et al., 2017, revealed that a high prevalence of depression associated with SLE often leads to high steroid use among the patients which further increases the BOD as it is associated with many side effects [29].

Recent advances in healthcare and the introduction of biological treatments for SLE seem promising in the management of the disease. However, these costly treatment options again pose a substantial economic burden. Understanding the burden of SLE in KSA is critical because of its unique genetic, environmental, and socio-cultural contexts. This study employed a COI model to estimate the clinical, humanistic, and economic impact associated with SLE. As the BOD studies comprehensively encapsulate the multifaceted impact of a disease on health outcomes across national, regional, community, and individual levels, these are helpful in prioritizing healthcare interventions and efficiently allocating resources within budget constraints [11]. It assesses the frequency of disease occurrence, its impact on life expectancy and morbidity, reductions in health status and quality of life (QOL), and the financial consequences [11].

## Methods

### Model structure

A COI model was developed using Microsoft Excel^®^ 365 from a societal perspective to estimate the costs and humanistic and economic burden of SLE on patients in the KSA. The analysis was done for a 1-year time horizon to minimize seasonal variability and as a best practice. Key model inputs included eligible population, treatment pattern, efficacy, and cost inputs, while the primary outcomes were the total costs and disease burden. Table 1 summarizes the parameters included in the model inputs. The analysis was done following the best practices of the International Society of Pharmacoeconomics and Outcomes Research (ISPOR).

**Table 1:** Speciality and institute type of the KOLs

Speciality	Number of experts	Institution type	Number of experts
Pharmacist	9	Academic Teaching Hospital	1
Internal medicine & Rheumatology	2	Government Health Authority	5
Clinical Pharmacy	2	Government Teaching Hospital	3
Internal medicine	1	Military Hospital	3
Internal Medicine Pharmacotherapy	1	Specialist & Research Hospital	2
Dermatology, Cosmetic	1	Tertiary Care Medical City	2
Rheumatology consultant	1	University Hospital	1

The data for all the model inputs were acquired from government databases, literature surveys, and key opinion leaders (KOL) opinions. In total, seventeen KOLs were invited, and the data were gathered through an offline survey (Supplementary information: Survey Questionnaire). The KOLs included were experienced clinicians and healthcare professionals actively involved in the care and management of SLE patients in the KSA for more than 10 years. Most of the KOLs have documented involvement in clinical guidelines or peer-reviewed publications, with several contributing specifically to national rheumatology and SLE guidelines—highlighting their relevance in shaping disease management strategies in the KSA (Table 1). All the information obtained from the KOLs is reported on an aggregate level in this study without disclosing individual identification.

### Model inputs

#### Epidemiology and patient population

This study did not enroll patients but included data from the government database for the population size of SLE patients who were more than 15 years of age with moderate to severe disease activity and were considered adults. The number of patients satisfying this criterion was estimated based on the country population database, literature survey, and KOLs opinions. Out of a total population of 32,175,224 of the KSA, 72.36% were above 15 years of age (https://www.stats.gov.sa/en). Based on the prevalence and incidence of SLE as estimated through literature sources, the total adult SLE patient population in the KSA was estimated to be around 5,216 at the time of this analysis [8, 30].

Moreover, based on the KOL, out of the total SLE adult patient population, 68.70% of people were clinically diagnosed with SLE and 92.50% underwent treatment. Additionally, 82.50% of the population consisted of patients with moderate to severe disease activity.

For indications like SLE, COI can vary depending on the disease severity and type. Therefore, in this study, we considered three types of SLE: SLE proper (systemic lupus involving multiple organs except renal and CNS), lupus nephritis, and lupus CNS.

Among the adult moderate to severe SLE patients, 1,331 patients were estimated to be affected by SLE proper, 1,574 with lupus nephritis, and 593 with lupus CNS, representing 38%, 45%, and 17% of the included population, respectively, as per the KOL opinion.

These patients were further categorized into autoantibody positive and autoantibody negative, as the treatment patterns differ between these groups which leads to variations in the total COI. According to KOLs opinion, 99.5% of people were estimated to be autoantibody positive, while less than1% were autoantibody negative.

#### Treatment pattern

The treatment pattern and management of SLE is an overlapping combination of various therapies, tailored according to the type and severity of the disease. The proportion of various therapies utilized for the treatment of antibody-positive and antibody-negative SLE proper, lupus nephritis, and lupus CNS was captured based on the real-world healthcare practice in the KSA utilizing the input gathered from the KOLs (Supplementary Material: Table 1S).

Among patients with SLE proper, 15% were estimated to be on biologics which included belimumab and anifrolumab, while 4% of those with lupus nephritis were estimated to be treated with belimumab along with the standard of care (SOC). Since neither of these drugs is used to treat lupus CNS, patients with this condition received only SOC (KOL opinion).

#### Efficacy

Clinical efficacy data of SOC and biological treatments for SLE and associated complications were gathered from published articles which are based on data gathered from various clinical trials. The efficacy was estimated in terms of response rate and belimumab was found to be slightly more effective with a 0.58 probability compared to anifrolumab with a probability of 0.42 [31] (Table 2).

**Table 2:** Model inputs

Parameter	Value							Source
**Population (N)**								
Country Population	32,175,224							GAStaat (https://www.stats.gov.sa/en)
Proportion of the population > 15 years of age	23,281,992 (72.36%)							
Prevalence of SLE	5,216 (0.02%)							[25]
Incidence of SLE	1,455 (0.006%)							[30]
Total population of SLE	6,671							KOL opinion
Patients Diagnosed	4,583 (68.70%)							
Patients Treated	4,239 (92.50%)							
Patients with moderate to severe disease activity	3,497 (82.50%)							
**SLE patient by Type (N)**								
SLE Proper	1,331							KOL opinion
Lupus Nephritis	1,574							
Lupus CNS	593							
**Autoantibody Positive Patients (N)**								
SLE Proper Autoantibody +	1,324							KOL opinion
Lupus Nephritis Autoantibody +	1,566							
Lupus CNS Autoantibody +	590							
**Autoantibody Negative Patients (N)**								
SLE Proper Autoantibody -	7							KOL opinion
Lupus Nephritis Autoantibody -	8							
Lupus CNS Autoantibody -	3							
**Proportion of patients on Biologics (N)**								
SLE Proper	15%							KOL opinion
Lupus Nephritis	4%							
**Efficacy**								
	**SOC**		**Belimumab**		**Anifrolumab**			
**SLE Index score (Relative risk reduction)**	-		1.67		1.60			[26]
Mucocutaneous Eruption/Ulceration	0.56		0.34		0.35			[27] The event rates are per patient per year. Considering that some patients might have more than one event per year and some patients will have zero events per year, the study considered the total events and divided them by the total number of patients to calculate the events per patient per year.
Alopecia	0.18		0.11		0.11			
Vasculitis	0.26		0.16		0.16			
Arthritis	0.56		0.34		0.35			
Pericarditis	0.33		0.20		0.21			
Pleuritis	0.36		0.22		0.23			
Anemia	0.07		0.04		0.04			
Thrombocytopenia	0.21		0.13		0.13			
Leucopenia	0.33		0.20		0.21			
Pyrexia	0.10		0.06		0.06			
SLE Nephritis (Nephrotic Syndrome)	1.00		0.60		NA			
SLE CNS	1.00		NA		NA			
**Response Rate**	32%		61%		47.8%			
**Events related disutility**								
Mucocutaneous Eruption/Ulceration	0.07							[27]
Alopecia	0.06							[28]
Vasculitis	0.05							[29]
Arthritis	0.09							[8]
Pericarditis	0.12							
Pleuritis	0.07							[30]
Anemia	0.01							
Thrombocytopenia	0.04							[31]
Leucopenia	0.04							
Pyrexia	0.08							
SLE Nephritis (Nephrotic Syndrome)	0.15							[32]
SLE CNS	0.0178							[33]
	**SOC**			**Belimumab**		**Anifrolumab**		
Mortality Rate	0.019			0.011		0.012	[34]	
Total Disutility (SLE Proper)	0.21			0.126		0.132		
Total Disutility (SLE Nephritis)	0.15			0.09		NA		
**Life Expectancy loss (years)**	10.80						GAStaat (https://www.stats.gov.sa/en)/	
**Per capita income loss (SAR)**	114,178.74						As per World Bank data of 2022	
**Adverse events rates**								
	**SOC**			**Belimumab**		**Anifrolumab**		
Injection/infusion site reaction	10.0%			12.0%		2.8%	Phase III study (HGS1006–C1115; BEL112341); EMA label	
Headache	7.0%			0.0%		6.0%		
Skin allergic reaction	7.0%			12.0%		0.1%		
Nasopharyngitis	5.0%			5.0%		8.0%		
Bronchitis	1.0%			3.0%		34.0%		
Cystitis	1.0%			3.0%		3.3%		
Herpes Zoster infection	1.3%			0.0%		0.0%		
**Event Management Costs (cost per event) (SAR)**								
Mucocutaneous Eruption/Ulceration	1,780						KOL opinion	
Alopecia	1,570							
Vasculitis	20,998							
Arthritis	8,642							
Pericarditis	7,222							
Pleuritis	5,141							
Anemia	4,497							
Thrombocytopenia	9,821							
Leucopenia	1,516							
Pyrexia	933							
Nephrotic Syndrome	18,756							
Neuropathy	3,204							

Abbreviations: EMA = European Medicines Agency = KOL = Key opinion leaders, SOC = Standard-of-care, SAR = Saudi Riyal, SLE = Systemic Lupus Erythematosus

DALYs were calculated as the sum of Years of Life Lost (YLL) and Years Lived with Disability (YLD). YLL was estimated using life expectancy data from the General Authority of Statistics (GASTAT), assuming a standard life expectancy at birth of 10.80 (https://www.stats.gov.sa/en). YLD was computed using disability weights from literature sources for different SLE related events (Table 1; Events related disutility). Mortality rates for indirect costs were monetized based on per capita income in KSA, which was SAR 114178.74 as per the World Bank for the reference year 2022. Direct medical costs and monitoring costs were derived from hospital billing data, published literature, including unit costs for outpatient visits and diagnostic tests. Utilization assumptions were based on expert opinion, and costs were applied to the healthcare settings from the perspective of KSA MOH.

Adverse events rates for SOC and biological treatments were gathered from the clinical trial data and European Medicines Agency (EMA) label (Table 2).

#### Costs

The COI studies traditionally stratify costs into three categories direct, indirect, and intangible costs [11]. Direct costs can be medical-related, including acquisition costs, administration and monitoring, conventional outpatient and inpatient procedures, and tests. They can also be non-medical-related, such as alternative care. Indirect cost includes productivity loss that specifically measures the burden of years of life lost (YLL) due to premature death and the years lost due to disability or morbidity (YLD). These two categories make up a measure of ‘cost’ called total DALYs (disability-adjusted life years), which encompass healthcare costs and the lost economic or societal contribution resulting from premature death or disability. Intangible assets include pain and suffering as well as disability.

The costs associated with drug acquisition, administration, monitoring, adverse events, and event management were calculated for SLE proper, lupus nephritis, and lupus CNS. These costs were estimated in Saudi Arab Riyal (SAR).

Drug acquisition and administration costs for the first year after SLE diagnosis and in the subsequent years were estimated for various treatment regimens for SLE based on the doses used and route of administration by exploring EMA/ Food and Drug Administration (FDA) labels of the respective interventions and gathering KOLs’ opinions (Table 3). Drug administration costs for SOC and biologicals were also estimated based on the KOLs’ opinions on the current clinical practice in the KSA.

**Table 3:** Drug acquisition, administration, and monitoring cost of Systemic Lupus Erythematosus in the Kingdom of Saudi Arabia

Regimen	Drug acquisition cost in first year (in SAR)	Drug acquisition cost in subsequent year (in SAR)	Drug administration cost in first year (in SAR)	Drug administration cost in subsequent year (in SAR)	Reference
Belimumab - SLE proper	38,049	38,049	25	0	EMA label
Belimumab - Lupus Nephritis	40,968	38,049	25	0	
Anifrolumab	39,353	39,353	16,946	16,946	
Rituximab	11,194	11,194	2600	2600	[35]
Hydroxychloroquine	545.4	509.0	0.0	0.0	EMA label/KOL opinion
Prednisolone	109	34.3335	0.0	0.0	
Dexamethasone	5	-	585	0	
Methylprednisolone	108	-	585	0	
Topical calcineurin	89	89	0	0	
Methotrexate	74	77	0	0	
Cyclophosphamide	72	-	0	0	
Leflunomide	1461	1461	0	0	
Azathioprine	923	923	0	0	
Ibuprofen	0.15	0.15	0	0	
Naproxen	237	237	0	0	
Warfarin	310	310	0	0	
Mycophenolate Mofetil	2,794	2,235	0	0	
Belimumab IV	33,990	30,482	18895.5	16945.5	FDA label
**Monitoring costs**	**Total costs**				
SOC	6506.86				KOL opinion
SOC+Belimumab	6506.86				
SOC+Anifrolumab	6506.86				

Abbreviations: EMA = European Medicines Agency, FDA = Food and Drug Administration, KOL = Key opinion leaders, IV = Intravenous, SOC = Standard-of-care

Disease monitoring costs for SOC and biologicals were captured separately in terms of physician charges and various diagnostic and monitoring tests based on the current clinical practice in the KSA and KOLs’ opinion.

Event management costs were determined based on the cost per event for various complications, including mucocutaneous eruptions/ulcerations, alopecia, vasculitis, pericarditis, arthritis, pleuritis, thrombocytopenia, pyrexia, anemia, and leukopenia. Adverse event costs were calculated for the reported adverse events related to the treatment with SOC and biologicals, such as injection site reactions, nasopharyngitis, herpes zoster infection, cystitis, bronchitis, headache, and skin allergic reactions.

### Model analysis

A One-Way Sensitivity Analysis (OWSA) in the Systemic Lupus Erythematosus (SLE) model was performed to assess the impact of uncertainty in individual parameters on the overall model outcomes. Each parameter was varied individually across a predefined range, while holding all other variables constant. Ranges were assumed to vary by ±10% from the base-case value. Parameters tested included patient population, drug efficacy, treatment pattern, treatment cost ad costs of events management and adverse events. In this case, parameters such as *New Patients each year (2024–2028)* and *Current Patients (2024–2028)* are varied independently to observe how changes in these inputs affect the total patient population or key outputs such as treatment cost, disease burden, or quality-adjusted life years (QALYs). The results were presented in a tornado diagram to identify which parameters had the greatest influence on the total burden.

### Validation by the Delphi panel

The data collected from the literature review and KOL interviews underwent further validation in a virtual roundtable discussion with a local Delphi panel of experts, that included esteemed clinicians and researchers from various medical and research centers across different regions of the country.

## Results

The BOD model for systemic lupus erythematosus (SLE) was developed over a 1-year time frame using Microsoft Excel as the analytical tool. The model incorporated a variety of inputs gathered through a comprehensive literature review and insights from KOLs. These inputs were further validated through a local Delphi panel to ensure the accuracy and relevance of the model assumptions. The primary objectives were to estimate the health outcomes and economic burden associated with SLE.

### Clinical burden for SLE

SLE is a complex and challenging autoimmune connective tissue disorder that can affect multiple organ systems. It is characterized by its diverse clinical manifestations including mucocutaneous eruption/ulceration, vasculitis, arthritis, pericarditis, pleuritis, and neuropathy. Furthermore, SLE is often associated with a range of autoimmune and inflammatory conditions, including hematological abnormalities such as anemia, thrombocytopenia, and leucopenia, which arise from the pathological effects of the disease on hematological parameters. Additionally, systemic symptoms like fever may indicate disease activity or infection, while renal involvement can lead to nephrotic syndrome due to lupus nephritis. Patients with SLE may also experience dermatological effects such as alopecia and neurological manifestations like peripheral neuropathy, adding to the complexity of the disease’s clinical presentation.

### Humanistic burden of SLE and the associated indirect cost

**Fig. 1:**
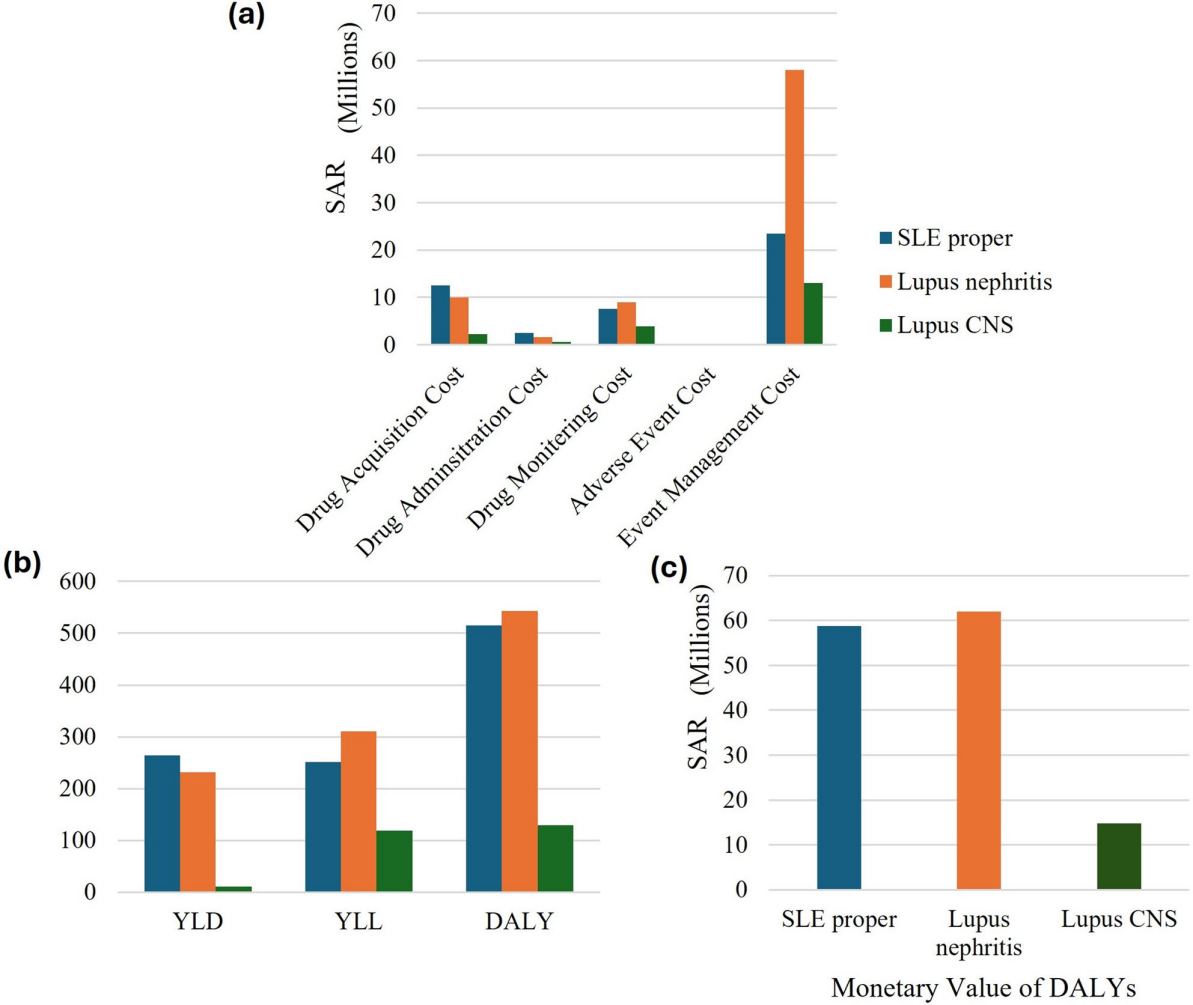
(**a**) Economic burden of systemic lupus erythematosus proper (SLE proper), lupus nephritis and lupus central nervous system (CNS) in terms of cost (including drug acquisition, administration, monitoring, adverse events, and events management). All costs are presented in Suadi Riyal (2024). Results are shown for total burden per year. The total cost is estimated at sar 46,187,056 for SLE proper, SAR 78,776,030 for lupus nephritis, and SAR 19,822,905 for lupus CNS. (**b**) and (**c**) monetary loss due to years lived with disability (YLD), years of life lost (YLL), and disability-adjusted life years (DALYs). Results are shown for total burden per year. SLE proper results in the DALYs of 514.70 with monitory value of SAR 58,768,258; lupus nephritis results in the DALYs of 542.96 with monitory value of SAR 31,854,460; and lupus CNS results in the DALYs of 129.67 with monitory value of SAR 14,805,541

The total number of DALYs lost due to SLE proper during the 1-year period was estimated at 514.7, driven by 263.6 years lived with disability (YLD) and 251.1 years of life lost (YLL), with an overall humanistic burden of SAR 58,768,258. On the other hand, for lupus nephritis, it was estimated to be 542.96 DALYs, comprising 232.12 YLD and 310.85 YLL, amounting to SAR 61,995,057 as the total monetary losses. For lupus CNS the model estimated 10.55 YLD and 119.12 YLL, totaling 129.67 DALY, which resulted in a total monetary loss of SAR 14,805,541 (Fig. 1). The model estimated an annual mortality rate of 23.25%, 28.78%, 11.03% among individuals with SLE proper, lupus nephritis and lupus CNS, respectively, significantly higher than the general population rate of 0.64% per year (Saudi Arabia (SAU) - Demographics, Health & Infant Mortality - UNICEF DATA).

### Economic burden of SLE due to direct cost

The overall economic burden of SLE proper, lupus nephritis and lupus CNS incurred by the direct expenses for one year was estimated to be amounted to SAR 46,187,056, 78,776,030, and 19,822,905 respectively. Event management cost was the primary contributor to the economic burden, followed by high drug acquisition costs, which also significantly add to overall healthcare expenses (Fig. 1).

### SOC versus biologicals: economic and humanistic impact

**Fig. 2:**
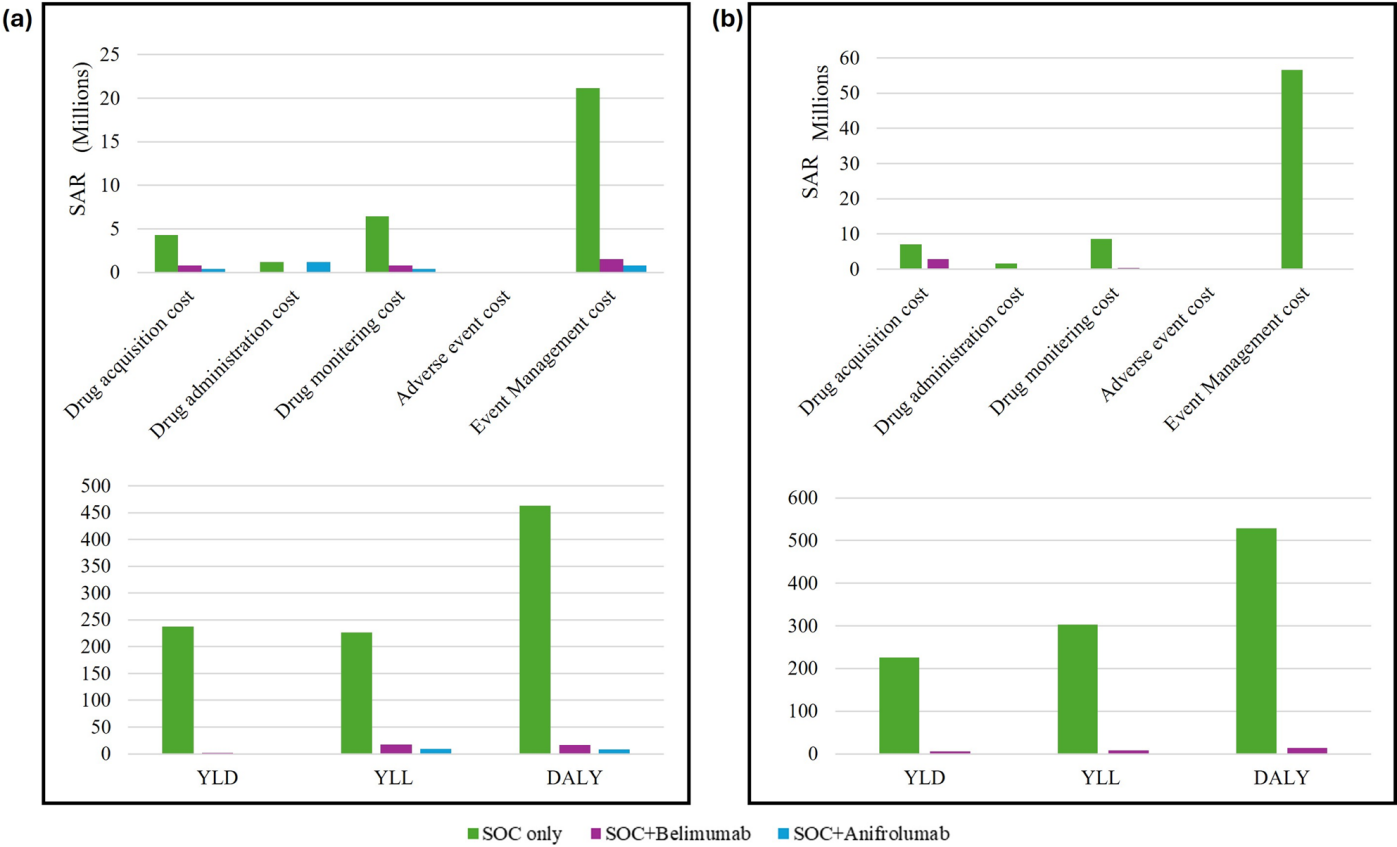
Comparison of the economic and humanistic burden of Systemic Lupus Erythematosus (SLE) in patients treated with standard-of-care versus biologicals (**a**) SLE proper; (**b**) Lupus nephritis). All costs are presented in Suadi Riyal (2024). Results are shown for total burden per year

The total drug acquisition costs and drug administration costs for SLE proper and lupus nephritis were higher for SOC compared to belimumab and anifrolumab. This cost difference is partly due to the larger number of patients treated under SOC- 1125 for SLE proper and 1508 for lupus nephritis—indicating a significant economic impact of patient volume on cost assessments. However, the per-patient cost of SOC was lower than that of biologics. There was little difference in drug monitoring costs for both treatment regimens. For SLE proper, the per-patient adverse event costs were lower for SOC combined with belimumab and higher for SOC combined with anifrolumab compared to SOC alone. In the case of lupus nephritis, the adverse event costs were higher for SOC in comparison to SOC combined with belimumab. The total and per-patient event management costs were higher for SOC in both SLE proper and lupus nephritis. Figure 2 shows the economic and humanistic burden of SLE when managed through SOC versus biological treatments.

**Fig. 3:**
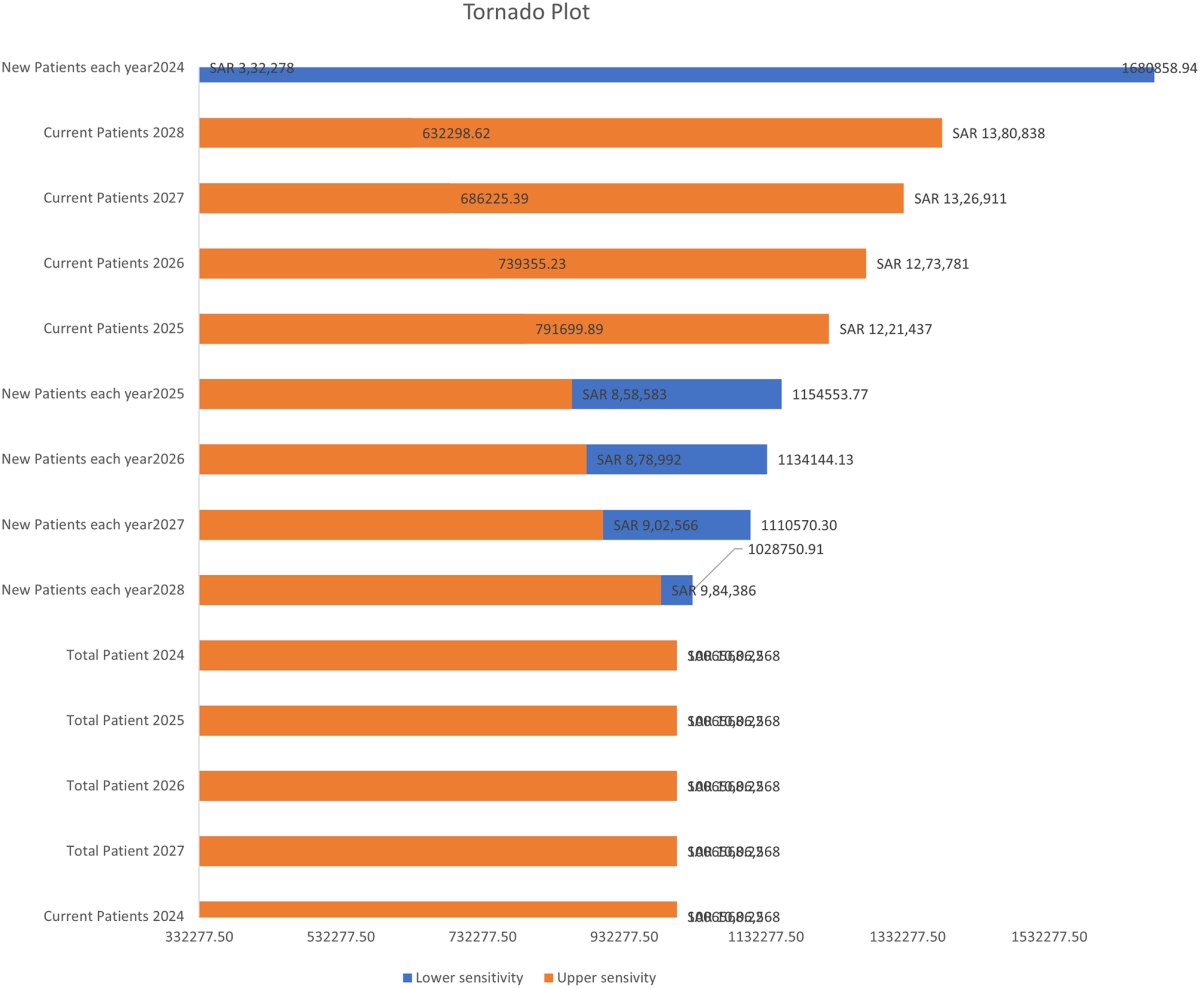
One-way sensitivity analysis: tornado graph

In OWSA, Current Patients 2024 group emerges as a major contributor, indicating that fluctuations in this parameter have the most significant influence on the model outcomes (Fig. 3). This suggests that the baseline patient pool is a critical driver of future disease prevalence and associated healthcare costs. This emphasizes the importance of accurate estimation and monitoring of the existing patient base when forecasting disease trends or evaluating interventions.

### Validation by the Delphi panel

The Delphi panel, comprised of esteemed clinicians and researchers from various medical and research centers across different regions of the country, was in agreement with the analysis. However, the panel recommended including people with mild disease activity in the COI analysis, as mild disease also has an economic and humanistic impact on the patient and society. These inputs from the Delphi panel indicate that the overall burden of SLE in the KSA is even more severe than what was analyzed in this study.

## Discussion

SLE is a chronic and multifaceted autoimmune disease characterized by a range of clinical symptoms that can vary from mild to severe [1, 36]. It primarily impacts young women of childbearing age and shows higher prevalence among African American, Hispanic, Asian, and Native American populations [35]. Despite advancements over time, SLE continues to significantly impact the quality of life, affecting both morbidity and mortality and consecutively affecting nation’s economy.

This study aimed to evaluate the clinical, economic, and humanistic burden of SLE in KSA. The data was gathered through a literature review and insights from KOLs, followed by validation from a Delphi panel of experts.

The economic burden of SLE revealed significant economic impacts within a one-year timeframe. The high cost associated with SLE proper can be attributed to the need for ongoing comprehensive management to control systemic inflammation and prevent organ damage.

Lupus nephritis posed an even greater economic impact which is likely due to its severe nature and the extensive treatment and management required to address renal complications. Moreover, lupus nephritis resulted in higher DALYs than SLE proper. This suggests that lupus nephritis incurs greater economic costs and humanistic burden in terms of DALYs than SLE proper. Aghdassi et al. also demonstrated that patients with lupus nephritis were more likely to see rheumatologists and nephrologists, undergo diagnostic tests, and incur higher medication costs [37].

These comparisons highlight the variability in economic and health impacts across different manifestations of SLE, emphasizing the need for targeted healthcare strategies to manage each condition effectively. The varying DALY values and economic costs associated with each condition provide crucial insights for healthcare policymakers to prioritize resources and interventions to mitigate the overall burden of SLE and its complications.

Alansari et al. (2024) also reported that the burden of SLE in the UAE is immense, primarily due to the expensive complications and productivity loss, as SLE predominantly affects individuals in their productive years [20]. Approximately 77% of SLE patients were shown to experience organ damage, making it a critical factor in SLE prognosis, as it significantly influences both morbidity and mortality [38, 39]. Kim et al. showed that increased healthcare costs were associated with disease severity, older age, major organ involvement, and comorbidities [40].

A real-world survey of SLE patients in the US and Europe found worse health, work productivity, tiredness, depression/anxiety, and emotional distress with increasing disease severity (*p* < 0.0001) [41]. SLE causes stress and anxiety in patients’ caregivers as it negatively impacts their presenteeism at work and in social life [42, 43]. The average duration of short-term sick leave was estimated to be between 7.0 and 64.8 days per year [43]. Inadequate social support is linked to higher disease activity and weakened mental performance [44].

BOD studies are critical tools in public health that quantify the impact of diseases and economic burden on populations. It aids in understanding comprehensive metrics for health impact, guiding policy, and resource allocation through detailed comparative and trend analyses. Moreover, OWSA plays a key role in validating the model and guiding decisions by highlighting parameters that may need better data or tighter control. In the SLE model, assessing sensitivity to the 2024 patient population helps refine projections, optimize resource use, and adapt treatment strategies to a changing patient landscape.

Nevertheless, this analysis may be subject to some limitations: data quality and completeness may vary across sources, potentially affecting the accuracy of burden estimates; health state valuations are subjective and may not fully reflect patient experiences; and projected costs are sensitive to assumptions and may vary across healthcare settings and over time.

## Conclusion

The prevalence of SLE poses a significant burden in KSA due to its high costs and productivity losses, particularly as it affects individuals during their productive years. Therefore, it is advisable to explore wider use of appropriate biological treatments to help reduce both clinical and economic impact. Increasing disease awareness and conducting further evaluations of these therapies are recommended to support more effective disease management within the Saudi population.

## Electronic supplementary material

Below is the link to the electronic supplementary material.

Supplementary Material 1

Supplementary Material 2

## Data Availability

No datasets were generated or analysed during the current study.
