# Supplementary material for: Clinical, humanistic, and economic burden of systemic lupus erythematosus in the Kingdom of Saudi Arabia
Source: Cost Eff Resour Alloc. 2025 Nov 25;23:71. doi: 10.1186/s12962-025-00678-w (PMC12681157; doi:10.1186/s12962-025-00678-w)

Supplementary Material

**Clinical, Humanistic, and Economic Burden of
Systemic Lupus Erythematosus in the Kingdom of Saudi Arabia**

Table 1S: Treatment Pattern

1. Without Biologicals


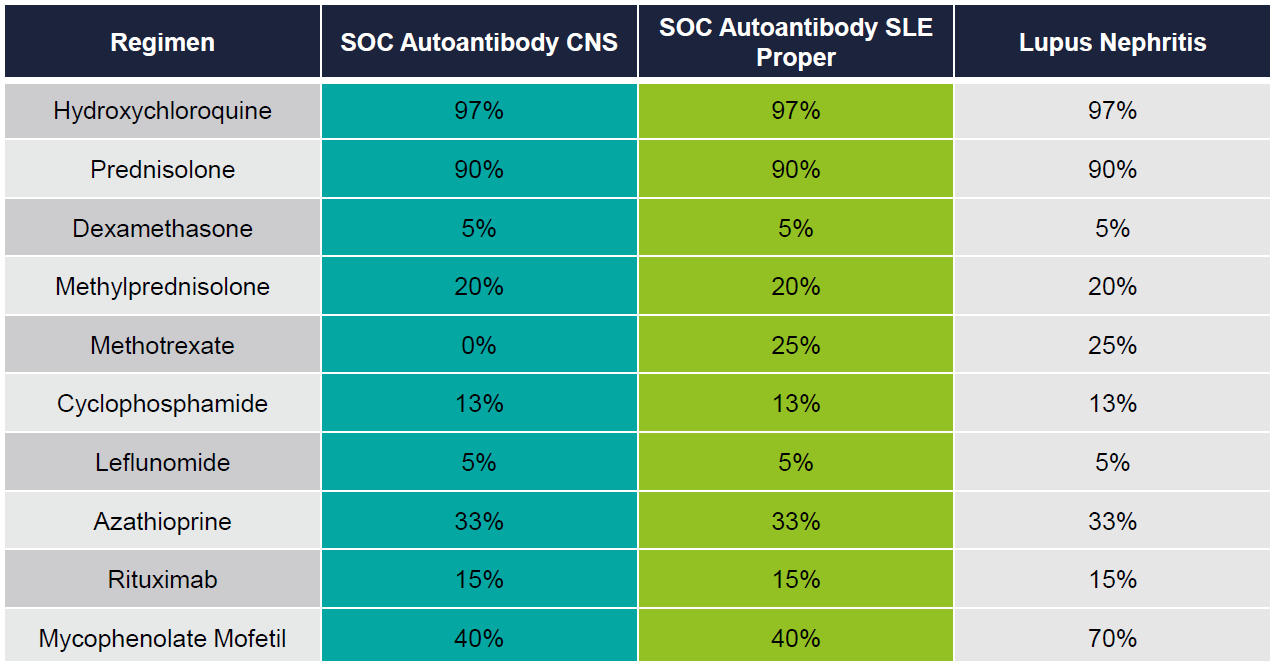


1. With Biologicals


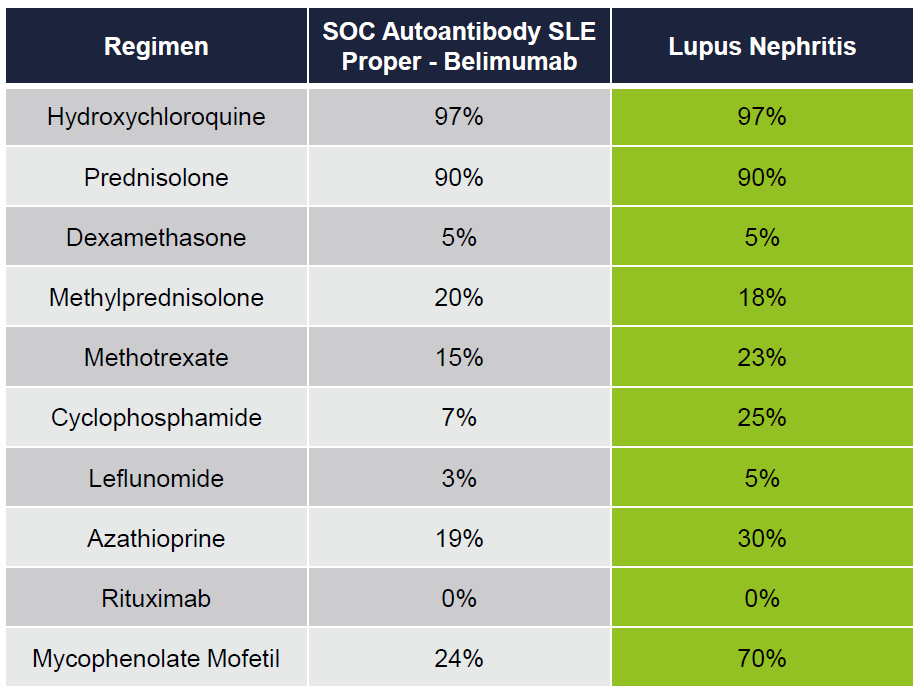

Supplement: Supplementary file 1 — Supplementary Material 1 [file 12962_2025_678_MOESM1_ESM.docx]
