# Supplementary material for: Clinical, humanistic, and economic burden of systemic lupus erythematosus in the Kingdom of Saudi Arabia
Source: Cost Eff Resour Alloc. 2025 Nov 25;23:71. doi: 10.1186/s12962-025-00678-w (PMC12681157; doi:10.1186/s12962-025-00678-w)
Supplement: Supplementary file 2 — Supplementary Material 2 [file 12962_2025_678_MOESM2_ESM.docx]

**Burden of disease of Systemic Lupus Erythematosus and Budget Impact Analysis of Biologicals for management of Systemic Lupus Erythematosus in Kingdom of Saudi Arabia from the Perspective of the Ministry of Health.**

*Confidential*

*The questionnaire remains the property of CareXso.*

| **INTRODUCTION** | |
| --- | --- |
| Good morning, my name is ______, from CAREXSO. We are conducting an in-depth interview on clinical practice in managing patients with systemic lupus erythematosus in your institute/hospital. All information will be reported on an aggregate level where no individual identification is disclosed.  Burden of disease of Systemic Lupus Erythematosus and Budget Impact Analysis of Biologicals for management of Systemic Lupus Erythematosus in Kingdom of Saudi Arabia from the Perspective of the Ministry of Health. The questionnaire is divided into different sections. The first section talks about the overview of systemic lupus erythematosus; in the next section we will talk about treatment options, dosing, and non-drug cost.  This interview will last for approximately 45-60 minutes. We may follow up with you after the interview for any outstanding information that is important for this project. Thank you for your kind assistance.  Abbreviations  KSA: Kingdom of Saudi Arabia  RRR- Relative Risk Reduction  SLE - Systemic Lupus Erythematosus   \| SECTION A: Overview \| \| --- \|  1. As per the multicentre cohort trial conducted in 2012 the regional prevalence of Systemic Lupus Erythematosus (SLE) in Saudi Arabia 19.28/100000. In your opinion, what is the current prevalence of SLE in KSA _________________%   Source: Almaghlouth, I.A. et al. (2021) ‘National systemic lupus erythematosus prospective cohort in Saudi Arabia’, Medicine, 100(30). doi:10.1097/md.0000000000026704.   1. As per the cohort study conducted the global incidence of SLE is 2 per 100000. In your opinion, what proportion of patients remain undiagnosed with SLE in KSA ________________%   Source: Study Group of Systemic Autoimmune Diseases (GEAS). Spanish Society of Internal Medicine (SEMI). Clinical Guidelines of Systemic Autoimmune Diseases. Disseminated Lupus Erythematosus. 2011. Available from: https://www.fesemi.org/sites/default/files/documentos/publicaciones/guia_les_2011.pdf. Accessed November 08, 2019.   1. In your clinical practice how many SLE patients do you examine per month?   Please provide the estimate ________   1. In your opinion, what is the proportion of SLE patients who would be diagnosed in MOH hospitals?   Proportion of patients _________%   1. In your opinion, what is the proportion of SLE patients who would seek treatment in MOH hospitals?   Proportion of patients _________%   1. In your opinion, what is the proportion of SLE patients have moderate to severe disease activity in KSA?   Proportion of patients _________%   1. As pe the Lupus Monitor around 59.29% of the SLE patients are autoantibody positive. In your opinion, what is the proportion of SLE patients are autoantibody positive KSA?   Proportion of patients _________%  Source: Ipsos Healthcare. Lupus Monitor market research 2016.   1. In your opinion, what is the proportion of total Lupus nephritis patients out of total SLE patients?   Please specify __________%   1. In your opinion, what is the proportion of total SLE Central Nervous system patients out of total SLE patients?   Please specify __________%   1. In your expert opinion what proportion of moderate to severe SLE patients are eligible for the biological therapy?   Please specify __________%   1. In your expert opinion what proportion of moderate to severe Lupus Nephritis patients are eligible for the biological therapy?   Please specify __________%   1. In your expert opinion what proportion of moderate to severe SLE central nervous system patients are eligible for the biological therapy?   Please specify __________%   1. In your opinion, what is clinical criteria do you use to assess the responsiveness to treatment of SLE patients?  \| Score \| Agree/ Disagree \| \| --- \| --- \| \| SLE Disease Activity Index 2000 (SLEDAI-2K) \|  \| \| British Isles Lupus Assessment Group (BILAG) \|  \| \| SLE Responder Index \|  \| \| Any other please specify \|  \|  \| SECTION B: Market Share \| \| --- \|   **In this section, we will discuss the management of moderate to severe SLE patients in your practice. Later, we would like to introduce two new drugs, Belimumab and Anifrolumab which has been approved for the management of Autoantibody positive moderate to severe SLE patients.**  Belimumab is a prescription medicine used to treat people with active systemic lupus erythematosus (SLE or lupus) or active lupus nephritis (lupus-related kidney inflammation) who are receiving other lupus medicines. It’s unknown if Belimumab is safe/effective in children under 5 years when given in a vein or under 18 years when given under the skin. Belimumab is not for people with severe active central nervous system lupus.  Anifrolumab is a type I interferon (IFN) receptor antagonist indicated for the treatment of adult patients with moderate to severe systemic lupus erythematosus (SLE), who are receiving standard therapy. Limitations of Use: The efficacy of SAPHNELO has not been evaluated in patients with severe active lupus nephritis or severe active central nervous system lupus. Use of SAPHNELO is not recommended in these situations.   1. ***Standard of care for the management of Moderate to severe SLE patients.*** 2. As per your clinical practice, how do you currently treat moderate to severe SLE patients? Please indicate the proportion of patients likely to receive these therapies. (Proportion of patients should sum up to be 100%)  \| Drugs \| Available in the KSA (Yes/No) \| Proportion of patients receiving the treatment \| \| --- \| --- \| --- \| \| Antimalarial \|  \|  \| \| - Hydroxychloroquine \|  \|  \| \| Corticosteroids \|  \|  \| \| - Prednisolone \|  \|  \| \| - Dexamethasone \|  \|  \| \| - Methylprednisolone \|  \|  \| \| Topical agents \|  \|  \| \| - Topical calcineurin \|  \|  \| \| Non-Biological Immunosuppressants \|  \|  \| \| - Methotrexate \|  \|  \| \| - Cyclophosphamide \|  \|  \| \| - Leflunomide \|  \|  \| \| - Azathioprine \|  \|  \| \| NSAID \|  \|  \| \| - Ibuprofen \|  \|  \| \| - Naproxen \|  \|  \| \| Biological Immunosuppressants \|  \|  \| \| - Rituximab \|  \|  \| \| Intravenous immunoglobulin \|  \|  \| \| Plasmapheresis \|  \|  \| \| - Any other ______please specify \|  \|  \| \| - Any other ______please specify \|  \|  \| \| - Total \|  \| 100% \|  1. ***Management of Moderate to severe autoantibody positive SLE (with no Lupus Nephritis/ CNS SLE) patients.*** 2. In your clinical opinion, dose the standard treatment pattern for the management of autoantibody positive SLE patients (with no Lupus Nephritis/ CNS SLE) differs from the management of non-autoantibody positive SLE patients? If yes, please specify the market share of standard line of management.  \| Drugs \| Proportion of patients receiving the treatment \| \| --- \| --- \| \| Antimalarial \|  \| \| - Hydroxychloroquine \|  \| \| Corticosteroids \|  \| \| - Prednisolone \|  \| \| - Dexamethasone \|  \| \| - Methylprednisolone \|  \| \| Non-Biological Immunosuppressants \|  \| \| - Methotrexate \|  \| \| - Cyclophosphamide \|  \| \| - Leflunomide \|  \| \| - Azathioprine \|  \| \| NSAID \|  \| \| - Ibuprofen \|  \| \| - Naproxen \|  \| \| Biological Immunosuppressants \|  \| \| - Rituximab \|  \| \| Intravenous immunoglobulin \|  \| \| Plasmapheresis \|  \| \| - Any other ______please specify \|  \| \| - Any other ______please specify \|  \| \| - Total \| 100% \|  1. Considering the biological like Belimumab and Anifrolumab are available in KSA for the prescription ins KSA.In your clinical opinion, what will be the new treatment pattern for the management of autoantibody positive SLE patients (with no Lupus Nephritis/ CNS SLE)? If yes, please specify the market share of new line of management.  \| Drugs \| Proportion of patients receiving the treatment \| \| \| \| \| \| --- \| --- \| --- \| --- \| --- \| --- \| \| 2024 \| 2025 \| 2026 \| 2027 \| 2028 \| \| Belimumab \|  \|  \|  \|  \|  \| \| Anifrolumab \|  \|  \|  \|  \|  \| \| Antimalarial \|  \|  \|  \|  \|  \| \| - Hydroxychloroquine \|  \|  \|  \|  \|  \| \| Corticosteroids \|  \|  \|  \|  \|  \| \| - Prednisolone \|  \|  \|  \|  \|  \| \| - Dexamethasone \|  \|  \|  \|  \|  \| \| - Methylprednisolone \|  \|  \|  \|  \|  \| \| Topical agents \|  \|  \|  \|  \|  \| \| - Topical calcineurin \|  \|  \|  \|  \|  \| \| Non-Biological Immunosuppressants \|  \|  \|  \|  \|  \| \| - Methotrexate \|  \|  \|  \|  \|  \| \| - Cyclophosphamide \|  \|  \|  \|  \|  \| \| - Leflunomide \|  \|  \|  \|  \|  \| \| - Azathioprine \|  \|  \|  \|  \|  \| \| NSAID \|  \|  \|  \|  \|  \| \| - Ibuprofen \|  \|  \|  \|  \|  \| \| - Naproxen \|  \|  \|  \|  \|  \| \| Biological Immunosuppressants \|  \|  \|  \|  \|  \| \| - Rituximab \|  \|  \|  \|  \|  \| \| Intravenous immunoglobulin \|  \|  \|  \|  \|  \| \| Plasmapheresis \|  \|  \|  \|  \|  \| \| - Any other ______please specify \|  \|  \|  \|  \|  \| \| - Any other ______please specify \|  \|  \|  \|  \|  \| \| - Total \| 100% \| 100% \| 100% \| 100% \| 100% \|  1. ***Management of Moderate to severe autoantibody positive Lupus Nephritis.*** 2. In your clinical opinion, dose the standard treatment pattern for the management of autoantibody positive Lupus Nephritis patients differs from the management of non-autoantibody positive SLE patients? If yes, please specify the market share of standard line of management.  \| Drugs \| Proportion of patients receiving the treatment \| \| --- \| --- \| \| Antimalarial \|  \| \| - Hydroxychloroquine \|  \| \| Corticosteroids \|  \| \| - Prednisolone \|  \| \| - Dexamethasone \|  \| \| - Methylprednisolone \|  \| \| Non-Biological Immunosuppressants \|  \| \| - Methotrexate \|  \| \| - Cyclophosphamide \|  \| \| - Leflunomide \|  \| \| - Azathioprine \|  \| \| NSAID \|  \| \| - Ibuprofen \|  \| \| - Naproxen \|  \| \| Biological Immunosuppressants \|  \| \| - Rituximab \|  \| \| Intravenous immunoglobulin \|  \| \| Plasmapheresis \|  \| \| - Any other ______please specify \|  \| \| - Any other ______please specify \|  \| \| - Total \| 100% \|  1. Considering the biological like Belimumab is available in KSA for the prescription ins KSA.In your clinical opinion, what will be the new treatment pattern for the management of autoantibody positive Lupus nephritis patients? Please specify the market share of new line of management.  \| Drugs \| Proportion of patients receiving the treatment \| \| \| \| \| \| --- \| --- \| --- \| --- \| --- \| --- \| \| 2024 \| 2025 \| 2026 \| 2027 \| 2028 \| \| Belimumab \|  \|  \|  \|  \|  \| \| Antimalarial \|  \|  \|  \|  \|  \| \| - Hydroxychloroquine \|  \|  \|  \|  \|  \| \| Corticosteroids \|  \|  \|  \|  \|  \| \| - Prednisolone \|  \|  \|  \|  \|  \| \| - Dexamethasone \|  \|  \|  \|  \|  \| \| - Methylprednisolone \|  \|  \|  \|  \|  \| \| Non-Biological Immunosuppressants \|  \|  \|  \|  \|  \| \| - Methotrexate \|  \|  \|  \|  \|  \| \| - Cyclophosphamide \|  \|  \|  \|  \|  \| \| - Leflunomide \|  \|  \|  \|  \|  \| \| - Azathioprine \|  \|  \|  \|  \|  \| \| NSAID \|  \|  \|  \|  \|  \| \| - Ibuprofen \|  \|  \|  \|  \|  \| \| - Naproxen \|  \|  \|  \|  \|  \| \| Biological Immunosuppressants \|  \|  \|  \|  \|  \| \| - Rituximab \|  \|  \|  \|  \|  \| \| Intravenous immunoglobulin \|  \|  \|  \|  \|  \| \| Plasmapheresis \|  \|  \|  \|  \|  \| \| - Any other ______please specify \|  \|  \|  \|  \|  \| \| - Any other ______please specify \|  \|  \|  \|  \|  \| \| - Total \| 100% \| 100% \| 100% \| 100% \| 100% \|  \| SECTION C: Treatment Practice \| \| --- \|   In this section, we will discuss about the treatment practice for the management of SLE patients.   1. Below table depicts the prescription practice for the management of SLE patients. Do you agree with the prescription practices? Also specify the proportion of patients on/ predicted to be on different maintenance doses.   (EMA Labels)   \| Regimen \| Dose (in mg) \| Dose (in mg) \| Frequency \| Duration \| Route of administration \| Agree/ Disagree \| \| --- \| --- \| --- \| --- \| --- \| --- \| --- \| \| Belimumab \| 200 mg \|  \| Once a week \|  \| SC \|  \| \| Anifrolumab \| 300 mg \|  \| Every 4 weeks \|  \| IV \|  \| \| Hydroxychloroquine \| 200 mg \|  \| once a day \|  \| Orally \|  \| \| 400 mg \|  \| once a day \|  \| Orally \|  \| \| Prednisolone \| 1 mg \|  \| once daily \|  \| Orally \|  \| \| 5 mg \|  \| once daily \|  \| Orally \|  \| \| 10 mg \|  \| once daily \|  \| Orally \|  \| \| 20 mg \|  \| once daily \|  \|  \|  \| \| Dexamethasone \| 8 mg \|  \| Once daily \|  \| IV \|  \| \| Methylprednisolone \| 100- 250 mg \|  \| Once daily \| 3-5 days \| IV \|  \| \| Methotrexate \| Starting with 2.5 mg and max do of 10 mg \|  \| once week \|  \| Orally \|  \| \| Cyclophosphamide \| 500 mg \|  \| Every alternate week \|  \| Orally \|  \| \| Leflunomide \| 100 mg \|  \| For 3 days \|  \| Orally \|  \| \| 10 -20 mg \|  \| Once daily \|  \| Orally \|  \| \| Azathioprine \| 2mg/kg \|  \| Once daily \|  \| Orally \|  \| \| Ibuprofen \| 100 -200 mg \|  \| Once daily \|  \| Orally \|  \| \| Naproxen \| 250-500 mg \|  \| Once daily \|  \| Orally \|  \| \| Rituximab \| 1 gm \|  \| On 1^st^ day then 15^th^ day \|  \| IV \|  \| \| Intravenous immunoglobulin \|  \|  \|  \|  \|  \|  \| \| Plasmapheresis \|  \|  \|  \|  \|  \|  \| \| Any other _________please specify \| ________ \|  \| ________ \|  \| ________ \| ________ \| \| Any other _________please specify \| ________ \|  \| ________ \|  \| ________ \| ________ \| | |

| **SECTION D: Major Complication Events** |
| --- |

In this section we will be asking you about the major complication events and its management. Please provide the management practices that you follow.

1. Below listed are complication/ Sequelae reported commonly in SLE patients.

We have mentioned the general services provided to manage these adverse events in the table below. Kindly mention whether you agree with the same? If not, how do you manage the adverse event in your patient?

If you are prescribing any additional medications or services to manage complication/ sequel, please add in table below:

1. **Mucocutaneous Eruption/ Ulceration**

| Treatment Protocol | Components | Dose | Frequency | Duration (Days) | Proportion of patients undergoing (%) |
| --- | --- | --- | --- | --- | --- |
| Dermatologist visits |  |  |  |  |  |
| Diagnostic tests | CBC |  |  |  |  |
| Medical management | Topical methylprednisolone |  |  |  |  |
|  | Topical betamethasone valerate |  |  |  |  |
|  | Topical Clobetasol |  |  |  |  |
|  | Tacrolimus 0.1%- 0.03% |  |  |  |  |
| Any other ________ (please specify) |  |  |  |  |  |
| Any other ________ (please specify) |  |  |  |  |  |

1. **Alopecia**

| Treatment Protocol | Components | Dose | Frequency | Duration (Days) | Proportion of patients undergoing (%) |
| --- | --- | --- | --- | --- | --- |
| Dermatologist visits |  |  |  |  |  |
| Diagnostic tests | CBC |  |  |  |  |
| Medical management | Iron supplémentation Ferrus sulfate Tablet (oral) | 200 mg |  |  |  |
|  | Minoxidil 0.5% (topical) |  |  |  |  |
| Any other ________ (please specify) |  |  |  |  |  |

1. **Vasculitis**

| Treatment Protocol | Components | Dose | Frequency | Duration (Days) | Proportion of patients undergoing (%) |
| --- | --- | --- | --- | --- | --- |
| Dermatologist visits |  |  |  |  |  |
| Diagnostic tests | CBC |  |  |  |  |
|  | ESR |  |  |  |  |
|  | CRP |  |  |  |  |
|  | Color Doppler |  |  |  |  |
|  | Culture and sensitivity |  |  |  |  |
| Medical management | Hydroxychloroquine (Oral) | 400 mg dose then reduce to 100 mg | Once daily |  |  |
|  | Thalidomide (Oral) | 100- 400 mg | Once daily |  |  |
|  | Dapsone (Oral) | 50 -100 mg | Once daily | 3-4 weeks |  |
|  | Prednisolone (Oral) | 1-2 mg | Once daily |  |  |
|  | Methylprednisolone is given intravenously 10 to 30 mg/kg (maximum 1 g) once  daily for 3 days. This is followed by 1 mg/kg/d of prednisolone orally, for 1 week,  and a gradual tapering over 1 month until the lowest maintenance dose is  achieved. | 10-30 mg/kg | Once daily |  |  |
|  | Intravenous immunoglobulins  High-dose (2.0 g/kg) IVIG |  |  | 3-5 month |  |
|  | Cyclophosphamide  1 to 3 mg/kg/d, and when the intravenous route is preferred, a suggested dosage of 0.5 to 1.0 g/m2 body surface area is given every 1 to  3 months. |  |  |  |  |
|  | Azathioprine  1 to 2.5 mg/kg/d in three divided doses or in equivalent intravenous |  |  |  |  |
|  | Methotrexate  The usual oral dosage is 7.5 to 10 mg/wk, which initially could be increased by 2.5  mg/wk to a total dosage of 20 mg/wk. |  |  |  |  |
|  | Cyclosporin A  orally in 1 to 3 mg/kg/d. |  |  |  |  |
| Hospitalization | In patient care |  |  |  |  |
|  | ICU care |  |  |  |  |
| Any other ________ (please specify) |  |  |  |  |  |

1. **Arthritis**

| Treatment Protocol | Components | Dose | Frequency | Duration (Days) | Proportion of patients undergoing (%) |
| --- | --- | --- | --- | --- | --- |
| Dermatologist visits |  |  |  |  |  |
| Diagnostic tests | CBC |  |  |  |  |
|  | ESR |  |  |  |  |
|  | CRP |  |  |  |  |
|  | Color Doppler |  |  |  |  |
|  | Culture and sensitivity |  |  |  |  |
|  | X ray long bone |  |  |  |  |
|  | CT scan of long bone |  |  |  |  |
|  | RA factor |  |  |  |  |
| Medical management | Hydroxychloroquine (Oral) | 400 mg dose then reduce to 100 mg | Once daily |  |  |
|  | Thalidomide (Oral) | 100- 400 mg | Once daily |  |  |
|  | Dapsone (Oral) | 50 -100 mg | Once daily | 3-4 weeks |  |
|  | Prednisolone (Oral) | 1-2 mg | Once daily |  |  |
|  | Methylprednisolone is given intravenously 10 to 30 mg/kg (maximum 1 g) once  daily for 3 days. This is followed by 1 mg/kg/d of prednisolone orally, for 1 week,  and a gradual tapering over 1 month until the lowest maintenance dose is  achieved. | 10-30 mg/kg | Once daily |  |  |
|  | Intravenous immunoglobulins  High-dose (2.0 g/kg) IVIG |  |  | 3-5 month |  |
|  | Cyclophosphamide  1 to 3 mg/kg/d, and when the intravenous route is preferred, a suggested dosage of 0.5 to 1.0 g/m2 body surface area is given every 1 to  3 months. |  |  |  |  |
|  | Azathioprine  1 to 2.5 mg/kg/d in three divided doses or in equivalent intravenous |  |  |  |  |
|  | Methotrexate  The usual oral dosage is 7.5 to 10 mg/wk, which initially could be increased by 2.5  mg/wk to a total dosage of 20 mg/wk. |  |  |  |  |
|  | Cyclosporin A  orally in 1 to 3 mg/kg/d. |  |  |  |  |
| Any other ________ (please specify) |  |  |  |  |  |

1. **Pleuritis**

| Treatment Protocol | Components | Dose | Frequency | Duration (Days) | Proportion of patients undergoing (%) |
| --- | --- | --- | --- | --- | --- |
| Pulmonologist visits |  |  |  |  |  |
| Diagnostic tests | CBC |  |  |  |  |
|  | ESR |  |  |  |  |
|  | CRP |  |  |  |  |
|  | Culture and sensitivity |  |  |  |  |
|  | X ray Chest |  |  |  |  |
|  | HRCT |  |  |  |  |
|  | Sputum examination |  |  |  |  |
| Medical management | Ibuprofen 100 -200 mg |  | Once daily |  |  |
|  | Naproxen 250-500 mg |  | Once daily |  |  |
|  | Prednisolone (Oral) | 1-2 mg | Once daily |  |  |
|  | Methylprednisolone is given intravenously 10 to 30 mg/kg (maximum 1 g) once  daily for 3 days. This is followed by 1 mg/kg/d of prednisolone orally, for 1 week,  and a gradual tapering over 1 month until the lowest maintenance dose is  achieved. | 10-30 mg/kg | Once daily |  |  |
| Hospitalisation | Emergency care visit |  |  |  |  |
|  | In Patient care |  |  |  |  |
|  | ICU Care |  |  |  |  |
| Any other ________ (please specify) |  |  |  |  |  |
| Any other ________ (please specify) |  |  |  |  |  |

1. **Pericarditis**

| Treatment Protocol | Components | Dose | Frequency | Duration (Days) | Proportion of patients undergoing (%) |
| --- | --- | --- | --- | --- | --- |
| Cardiologist visits |  |  |  |  |  |
| Diagnostic tests | CBC |  |  |  |  |
|  | ESR |  |  |  |  |
|  | CRP |  |  |  |  |
|  | ECG |  |  |  |  |
|  | 2 d Echo |  |  |  |  |
|  | X ray Chest |  |  |  |  |
|  | Cardiac CT |  |  |  |  |
|  | Cardiac MRI |  |  |  |  |
| Medical management | Ibuprofen 100 -200 mg |  | Once daily |  |  |
|  | Naproxen 250-500 mg |  | Once daily |  |  |
|  | Prednisolone (Oral) | 1-2 mg | Once daily |  |  |
|  | Methylprednisolone is given intravenously 10 to 30 mg/kg (maximum 1 g) once  daily for 3 days. This is followed by 1 mg/kg/d of prednisolone orally, for 1 week,  and a gradual tapering over 1 month until the lowest maintenance dose is achieved. | 10-30 mg/kg | Once daily |  |  |
|  | Colchicine 0.5 mg once a day |  |  |  |  |
| Hospitalisation | Emergency care |  |  |  |  |
|  | In patient care |  |  |  |  |
|  | ICU care |  |  |  |  |
| Any other ________ (please specify) |  |  |  |  |  |

1. **Neuropathy**

| Treatment Protocol | Components | Dose | Frequency | Duration (Days) | Proportion of patients undergoing (%) |
| --- | --- | --- | --- | --- | --- |
| Neurophysician visits |  |  |  |  |  |
| Diagnostic tests | CBC |  |  |  |  |
|  | Nerve conduction study |  |  |  |  |
|  | MRI Brain |  |  |  |  |
| Medical management | Thalidomide (Oral) | 100- 400 mg | Once daily |  |  |
|  | Dapsone (Oral) | 50 -100 mg | Once daily | 3-4 weeks |  |
|  | Prednisolone (Oral) | 1-2 mg | Once daily |  |  |
|  | Methylprednisolone is given intravenously 10 to 30 mg/kg (maximum 1 g) once  daily for 3 days. This is followed by 1 mg/kg/d of prednisolone orally, for 1 week,  and a gradual tapering over 1 month until the lowest maintenance dose is  achieved. | 10-30 mg/kg | Once daily |  |  |
|  | Cyclophosphamide  1 to 3 mg/kg/d, and when the intravenous route is preferred, a suggested dosage of 0.5 to 1.0 g/m2 body surface area is given every 1 to  3 months. |  |  |  |  |
|  | Azathioprine  1 to 2.5 mg/kg/d in three divided doses or in equivalent intravenous |  |  |  |  |
|  | Methotrexate  The usual oral dosage is 7.5 to 10 mg/wk, which initially could be increased by 2.5  mg/wk to a total dosage of 20 mg/wk. |  |  |  |  |
|  | Cyclosporin A  orally in 1 to 3 mg/kg/d. |  |  |  |  |
| Hospitalization | In patient care |  |  |  |  |
| Any other ________ (please specify) |  |  |  |  |  |

1. **Anaemia**

| Treatment Protocol | Components | Dose | Frequency | Duration (Days) | Proportion of patients undergoing (%) |
| --- | --- | --- | --- | --- | --- |
| Haematologist visits |  |  |  |  |  |
| Diagnostic tests | CBC |  |  |  |  |
|  | Serum ferritin |  |  |  |  |
|  | Peripheral Smear Examination |  |  |  |  |
| Medical management | Iron supplémentation Ferrus sulfate Tablet (oral) | 200 mg | Once daily |  |  |
| Hospitalization | In patient care |  |  |  |  |
| Any other ________ (please specify) |  |  |  |  |  |
| Any other ________ (please specify) |  |  |  |  |  |

1. **Thrombocytopenia**

| Treatment Protocol | Components | Dose | Frequency | Duration (Days) | Proportion of patients undergoing (%) |
| --- | --- | --- | --- | --- | --- |
| Haematologist visits |  |  |  |  |  |
| Diagnostic tests | CBC |  |  |  |  |
|  | Serum ferritin |  |  |  |  |
|  | Peripheral Smear Examination |  |  |  |  |
| Medical management | Platelet transfusion |  |  |  |  |
|  | Prednisolone (Oral) |  |  |  |  |
|  | Methylprednisolone is given intravenously 10 to 30 mg/kg (maximum 1 g) once  daily for 3 days. This is followed by 1 mg/kg/d of prednisolone orally, for 1 week,  and a gradual tapering over 1 month until the lowest maintenance dose is  achieved. |  |  |  |  |
| Hospitalization | In patient care |  |  |  |  |
| Any other ________ (please specify) |  |  |  |  |  |
| Any other ________ (please specify) |  |  |  |  |  |

1. **Leukopenia**

| Treatment Protocol | Components | Dose | Frequency | Duration (Days) | Proportion of patients undergoing (%) |
| --- | --- | --- | --- | --- | --- |
| Haematologist visits |  |  |  |  |  |
| Diagnostic tests | CBC |  |  |  |  |
|  | X ray Chest |  |  |  |  |
|  | Peripheral Smear Examination |  |  |  |  |
|  | Urine examination |  |  |  |  |
| Medical management | Oral Cefixime (Oral) | 200 mg | 2 times a day | 5 days |  |
| Any other ________ (please specify) |  |  |  |  |  |
| Any other ________ (please specify) |  |  |  |  |  |

1. **Pyrexia**

| Treatment Protocol | Components | Dose | Frequency | Duration (Days) | Proportion of patients undergoing (%) |
| --- | --- | --- | --- | --- | --- |
| Haematologist visits |  |  |  |  |  |
| Diagnostic tests | CBC |  |  |  |  |
|  | X ray Chest |  |  |  |  |
|  | Peripheral Smear Examination |  |  |  |  |
|  | Urine examination |  |  |  |  |
| Medical management | Oral Céfixime (Oral) | 200 mg | 2 times a day | 5 days |  |
| Hospitalization | Emergency care |  |  |  |  |
|  | In patient care |  |  |  |  |
| Any other ________ (please specify) |  |  |  |  |  |
| Any other ________ (please specify) |  |  |  |  |  |

1. **Nephrotic Syndrome**

| Treatment Protocol | Components | Dose | Frequency | Duration (Days) | Proportion of patients undergoing (%) |
| --- | --- | --- | --- | --- | --- |
| Nephrologist visits |  |  |  |  |  |
| Diagnostic tests | Urine Albumin level |  |  |  |  |
|  | Albumin/creatinine ratio |  |  |  |  |
|  | Glomerular filtration rate (GFR) |  |  |  |  |
|  | CT KUB |  |  |  |  |
|  | MRI KUB |  |  |  |  |
|  | Kidney biopsy |  |  |  |  |
| Medical management | Angiotensin-converting enzyme (ACE) Inhibitors  Please specify________ |  |  |  |  |
|  | Angiotensin 2 receptor blockers (ARBs) In  Please specify_________ |  |  |  |  |
|  | Statin drugs  Please specify _________ |  |  |  |  |
|  | Finerenone |  |  |  |  |
| Kidney Dialysis |  |  |  |  |  |
| Kidney transplant |  |  |  |  |  |
| Please clarify the number of days of stay in regular ward/ ICU for medical management |  |  |  |  |  |
| Please clarify the number of days of stay in regular ward/ ICU for dialysis |  |  |  |  |  |
| Please clarify the number of days of stay in regular ward/ ICU for kidney transplant. |  |  |  |  |  |
| Any other ________ (please specify) |  |  |  |  |  |
| Any other ________ (please specify) |  |  |  |  |  |

| **SECTION E: Adverse Events** |
| --- |

In this section, we will discuss healthcare resource utilization and the associated costs in the management of adverse events associated with different treatment regimen for SLE patients.

1. Below listed are adverse events reported commonly in SLE patients on different medications based on clinical trials.

We have mentioned the general services provided to manage these adverse events in the table below. Kindly mention whether you agree with the same? If not, how do you manage the adverse event in your patient?

If you are prescribing any additional medications or services to manage adverse events, please add in table below: -

| Adverse event | Services provided to manage the AE | Proportion of patients | Duration of treatment |
| --- | --- | --- | --- |
| Vomiting /Nausea | Medicines like serotonin antagonist (ondansetron, granisetron) etc. |  |  |
| Diarrhoea | Medicines like loperamide, electrolyte replacement solutions, bifilac etc.  (*Please specify the name of medicine) _________*  *Additional services, if any (please specify)*  *________________*  *_______________* |  |  |
| Abdominal pain | Medications ______  (*Please mention the names of the medications*)  __________ |  |  |
| Fatigue | No medication  *Any treatment that you give (please specify)*  *____________*  *_____________* |  |  |
| Injection site reaction | Cold foamatation  Antihistamines _________ |  |  |
| Hypersensitivity | IV fluids  IV Steroids________  Antihistamines _________  Bronchodilators_________  Vasodilators____________  Hospitalization  ICU stays  Ward stays |  |  |
| Headache | Medications ______  (*Please mention the names of the medications*)  __________ |  |  |
| Skin allergic reaction to injection | IV Steroids________ |  |  |
| Rash | Dermatologist consultation  Additional services, if any (please specify)  Medication_______  (Please mention the names of the medications) |  |  |
| Nasopharyngitis | General physician visit  Tab Amoxicillin Clavulanates 625 mg 3 times a day for 5 days |  |  |
| Bronchitis | General physician visit  Tab Amoxicillin Clavulanates 625 mg 3 times a day for 5 days  Tab Salbutamol |  |  |
| Cystitis | General physician visit  Tab Norfloxacin 400 mg two times a day for 5 days |  |  |
| Herpes Zoster Infection | General physician visit  Acyclovir 800 mg orally five times daily for 7 to 10 days  Famciclovir 500 mg orally three times daily for 7 days  Valacyclovir 1,000 mg orally three times daily for 7 days  Prednisone 30 mg orally twice daily on days 1 through 7; then 15 mg twice daily on days 8 through 14; then 7.5 mg twice daily on days 15 through 21 2 (2 to 4) for days 1 through 7 2 (1 to 3) for days 8 through 14 1 (1 to 2) for days 15 to 21 |  |  |
| Any other _________please specify |  |  |  |
| Any other _________please specify |  |  |  |

| **SECTION F: Monitoring cost** |
| --- |

In this section, we will discuss healthcare resource utilization and the associated costs in the monitoring and follow up associated with different treatment regimen for Diabetes Mellitus patients.

1. Below listed are monitoring tests commonly conducted in SLE patients on standard of care based on clinical trials. Please specify the proportion of patients and frequency of tests conducted.

| Monitoring tests | Proportion of patients | Frequency per year |
| --- | --- | --- |
| dsDNA antibody tests | Please specify________ | Please specify________ |
| Atiphospholipid tests | Please specify________ | Please specify________ |
| anti-SSA (Ro) tests | Please specify________ | Please specify________ |
| anti-SSB (La) tests | Please specify________ | Please specify________ |
| anti-RNP antibody tests | Please specify________ | Please specify________ |
| direct Coombs tests | Please specify________ | Please specify________ |
| thyroid function tests | Please specify________ | Please specify________ |
| C1q binding and anti-C1q IgG antibody assays | Please specify________ | Please specify________ |
| Any other Please specify________ |  |  |
| Any other Please specify________ |  |  |

1. Below listed are monitoring tests commonly conducted in SLE patients on belimumab based on clinical trials. Please specify the proportion of patients and frequency of tests conducted.

| Monitoring tests | Proportion of patients | Frequency per year |
| --- | --- | --- |
| dsDNA antibody tests | Please specify________ | Please specify________ |
| Atiphospholipid tests | Please specify________ | Please specify________ |
| anti-SSA (Ro) tests | Please specify________ | Please specify________ |
| anti-SSB (La) tests | Please specify________ | Please specify________ |
| anti-RNP antibody tests | Please specify________ | Please specify________ |
| direct Coombs tests | Please specify________ | Please specify________ |
| thyroid function tests | Please specify________ | Please specify________ |
| C1q binding and anti-C1q IgG antibody assays | Please specify________ | Please specify________ |
| Any other Please specify________ |  |  |
| Any other Please specify________ |  |  |

1. Below listed are monitoring tests commonly conducted in SLE patients on Anifrolumab based on clinical trials. Please specify the proportion of patients and frequency of tests conducted.

| Monitoring tests | Proportion of patients | Frequency per year |
| --- | --- | --- |
| dsDNA antibody tests | Please specify________ | Please specify________ |
| Atiphospholipid tests | Please specify________ | Please specify________ |
| anti-SSA (Ro) tests | Please specify________ | Please specify________ |
| anti-SSB (La) tests | Please specify________ | Please specify________ |
| anti-RNP antibody tests | Please specify________ | Please specify________ |
| direct Coombs tests | Please specify________ | Please specify________ |
| thyroid function tests | Please specify________ | Please specify________ |
| C1q binding and anti-C1q IgG antibody assays | Please specify________ | Please specify________ |
| Any other Please specify________ |  |  |
| Any other Please specify________ |  |  |

END-

Thank you for your time today and taking part in this discussion
